# Optimal care for the management of older people non-weight bearing after lower limb fracture: a consensus study

**DOI:** 10.1186/s12877-021-02265-z

**Published:** 2021-05-24

**Authors:** S. Aloraibi, J. Gladman, D. Godfrey, V. Booth, K. Robinson, E. Lunt, A. Caswell, M. Kerr, B. Ollivere, A. L. Gordon

**Affiliations:** 1 Division of Rehabilitation, Ageing and Wellbeing, The Medical School, Queens Medical Centre, University of Nottingham, B109, Floor B, Derby Road, Nottingham, NG7 2UH UK; 2 Division of Rehabilitation, Ageing and Wellbeing, NIHR Nottingham Biomedical Research Centre, Medical School, QMC, B111, Nottingham, NG7 2UH UK; 3 Queen’s Medical Centre, Nottingham University Hospitals NHS Trust, Room WC1388 Level C, West Block, Nottingham, NG7 2UH UK; 4 NHR Applied Research Collaboration (ARC) East Midlands, Nottingham, UK; 5 Nottinghamshire Healthcare NHS Foundation Trust, Lings Bar Hospital, Beckside, Gamston, Nottingham, NG2 6PR UK; 6 Royal Derby Hospital, University Hospitals of Derby and Burton NHS Foundation Trust, Derby Medical School, Room DSN407, Derby, DE22 3NE UK

**Keywords:** Lower limb fractures, Non-weight bearing, Delphi process, Old people

## Abstract

**Background:**

Older people who are non-weight-bearing after a lower limb fracture are at risk of poor outcomes but there are no clinical guidelines for this group of patients. Given the paucity of the research evidence base, we conducted a consensus exercise to ascertain expert opinion about the management of this group.

**Methods:**

A three-round e-Delphi technique was planned to use the online JISC survey tool with a multidisciplinary panel of health professionals. Panellists were invited by email via professional organisations and UK NHS Trusts. The initial statements for this study were prepared by the authors based upon the findings of their scoping review. Consensus required >/= 70% agreement with statements.

**Results:**

Only 2 survey rounds were required. Ninety panellists, representing seven clinical disciplines, reached consensus for 24 statements about general issues (osteoporosis detection and management, falls risk reduction and nutrition) and specific non-weight bearing issues (such as the need for activity to be promoted during this period).

**Conclusions:**

These findings can be used in the generation of a clinical guideline for this group of patients.

**Supplementary Information:**

The online version contains supplementary material available at 10.1186/s12877-021-02265-z.

## Background

Lower limb fractures may result in substantial morbidity and mortality [1, 2]. Some patients with lower limb fractures are advised to limit the amount of weight they put through the affected limb until it is sufficiently healed – particularly where bone quality is poor. Restrictions can be non-weight bearing or partial-weight bearing and are typically for 6–12 weeks [3–6]. These restrictions can lead to immobility, dependency, and lengthy hospital admissions, particularly in older people who are vulnerable due to co-pathology and have limited functional reserves [7–10].

Our systematic scoping review of the literature concerning the management of people during a period of weight bearing restrictions after lower limb fractures showed that the patient group has not been well-studied and that there are no relevant specific clinical guidelines (Aloraibi S, Booth V, Robinson KR, Lunt EK, Godfrey D, Caswell A, et al: Optimal management of older people with frailty non-weight bearing after lower limb fracture: a scoping review. Age Ageing, forthcoming). Without a clinical guideline, it is difficult to assure high quality care for these patients or ensure optimal care when evaluating novel interventions to improve outcomes. Our review found evidence that could contribute to the development of a guideline: observational studies showed that depression, cognitive impairment and malnutrition adversely affected outcome; expert personal commentaries emphasised the importance of maintaining strength and range of movement during immobilisation, and the application of an orthogeriatric model of care. We found 111 guidelines regarding the management of fragility fractures and osteoporosis which are likely to apply to many but not all in this patient group. However, we found no consensus studies of expert opinion and practice specific to patients with weight-bearing restrictions after lower limb fracture. We report here such a consensus study, undertaken as a step towards developing a clinical guideline based upon best evidence and best current practice.

## Methods

We used a modified Delphi technique [11], a recommended method for when the evidence base is limited yet there is a considerable amount of tacit professional experiential knowledge [12–16]. We report this study in line with recent reporting recommendations [12]. The protocol has been published [17]. The University of Nottingham Faculty of Medicine and Health Sciences Research Ethics Committee approved the study [reference number: 423–1911].

We aimed to include a range of healthcare professionals with experience of, and interest in, the management of this patient group. Our research group included PPI members who felt that it was appropriate to seek consensus solely amongst healthcare professionals, given the highly technical nature of the domains around which consensus was being sought. Accordingly, we sought panellists who were geriatric medicine consultants, orthopaedic surgeon consultants, registered nurses, specialist physiotherapists and occupational therapists, clinical dieticians, and pharmacists.

We sought panellists by emails sent to UK professional organisations (*n* = 27) and a sample of relevant clinical departments of NHS Trusts in England (*n* = 17), requesting dissemination of the invitation to individuals on their mailing lists (Additional file 1). The invitation requested interested individuals to follow a link to the on-line survey.

To minimise respondent burden and hence optimise participation, we collected limited information about panellists, recording their professional group, their specialities, education level and length of experience. A declaration was required that they had experience of this patient group and had no significant financial conflict of interests. Completion of these details was taken as consent to participate.

We generated 24 initial statements for this study, based upon the findings of our scoping review and clinical experience. The study team (including clinically active geriatricians, an orthopaedic surgeon, a nurse, physiotherapists, an occupational therapist and our patient and public involvement team) informally generated these statements via discussions with each other, and with clinical dietetic and pharmacy colleagues and our patients and public involvement team (Additional file 1).

The study was conducted using the JISC (formerly Bristol) online survey tool (https://www.onlinesurveys.ac.uk/). The provisional statements were piloted and adjusted using the JISC platform using a convenience sample of panellists covering the range of the study target groups.

Each panellist was asked to select either ‘agree’ or ‘disagree’ responses to each statement in the on-line survey. Consensus was assumed if > 70% panellists agreed with a statement: there is no agreed percentage for Delphi studies but this is a value between the highest and lowest reported in a methodological review [18]. We calculated that a sample size of 80 responses in the final round would ensure that a statement achieving this level of consensus (> 70%) would have a 95% probability of reflecting a majority (> 50%). Allowing for drop out, we aimed to recruit 120 panellists.

A three survey round Delphi process was planned. Panellists’ responses were analysed after each survey round. Statements achieving consensus were removed from subsequent survey rounds. Statements not achieving consensus agreement (including those with consensus disagreement) at each survey round were reviewed by the study team, modified taking into account panellists’ free text responses and included into subsequent rounds. After all rounds, statements achieving and not achieving consensus were reported.

## Results

The study took place between August and November 2020. We exceeded our target sample size of 80 respondents for the final round by achieving 90 panellists in our second and final round. Despite inviting participants from UK-based organisations, the invitations were shared with their international members and panellists came from USA, Canada, Australia, Europe and the UK.

Nineteen of the initial 24 statements achieved consensus in survey round 1. In survey round 1 we deliberately included two opposing statements regarding the use of Vitamin D, one of which achieved consensus (levels should be checked before giving Vitamin D) and one which was rejected (Vitamin D should be given without checking levels). We produced eight further statements based on the four that did not achieve consensus and revised the Vitamin D statement that had achieved consensus, in view of panellists’ suggestions and entered it into survey round 2. These nine formed the statements for survey round 2. In survey round 2, six achieved consensus (Table 1: Statements 1.6–1.11). After we analysed the results of round 2 we decided not to proceed to a third survey round because the statements that had not achieved consensus were the reverse of statements that had achieved consensus. Thus, our study produced 24 consensus statements. One hundred and fifteen panellists participated in round 1 and 90 participated in round 2, representing seven different disciplines (Table 2).

**Table 1:** Statements in each round and level of agreement

Domains	Statements (round, % agreement)
Generic	1.1 Optimal care for the management of older people with frailty, non-weight bearing after lower limb fracture should comply with current national guidelines for the care of patients with fragility fractures with respect to osteoporosis detection and management; falls risk reduction, and nutrition (R1, 98%). 1.2. Irrespective of the place of care, patients require access to a multi-professional team including orthopaedic surgeons and physicians, nurses and rehabilitation professionals with expertise in geriatric care. They will take an approach based on, or compatible with, comprehensive geriatric assessment – covering 1.3a symptoms 1.3b physical functioning 1.3c continence 1.3d activity – previous and current, personal and instrumental ADL 1.3e management of co-pathology beyond osteoporosis 1.3f skin integrity 1.3 g a medication review 1.3 h cognition 1.3i affect 1.3j social network 1.3 k environment 1.3 l personal factors (e.g. religious or cultural needs/ requirements) (R1, 98%). 1.3. Inpatient care for these patients should be via an orthogeriatric service (R1, 95%). 1.4. Osteoporosis management and falls risk reduction should be co-ordinated by a fracture liaison service (R1, 84%). 1.5. The daily requirements for protein, calories, vitamins and other vital nutrients for each individual patient should be estimated, and a dietary plan to meet those requirements produced (R1 90%). 1.6 Vitamin D levels should be checked in all patients, and corrected if abnormal, according to locally agreed guidelines (R2, 89%). 1.7. Nutritional supplements should not be routinely offered (R2, 74%). 1.8. Nutritional supplements should only be offered to individual patients after a clinical assessment indicates nutritional needs that cannot be met by dietary means alone (R2, 86%) 1.9. Protein supplementation should only be offered to individual patients after a clinical assessment indicates a need that cannot be met by dietary means alone (R2, 83%). 1.10. Calorific supplements should only be offered to individual patients after a clinical assessment indicates nutritional needs that cannot be met by dietary means alone (R2, 85%). 1.11. Multivitamin supplements should only be offered to individual patients after a clinical assessment indicates nutritional needs that cannot be met by dietary means alone (R2, 77%).
2.Non-weight bearing period	2.1. At the onset of the period of NWB, a personalised programme of activity and exercise should be devised, agreed and recorded: 2.1a to reduce sedentary behaviour 2.1b to include a daily range of motion exercises for the lower limb joints 2.1c to include daily aerobic fitness exercises 2.1d to include daily strength exercises for all limbs (R1,98%). 2.2. At onset of the period of NWB, thromboembolism prevention management should be reviewed, and should comprise 2.2a mobilization 2.2b mechanical (e.g. compression hosiery if tolerated) 2.2c low dose heparinoid unless contraindicated (R1, 96%). 2.3. At onset of the period of NWB, plans for the monitoring and management of any wound, fixation device or limb casting during the period of non-weight bearing should be recorded (R1,99%). 2.4. At the onset of the period of NWB, plans for the duration of the period of non-weight bearing, or the decision-making process to define it, should be recorded (R1,99%). 2.5. At the onset of the period of NWB, specific plans for the consequences of the personal ADL limitations imposed by the requirement for non-weight bearing such as upon skincare, continence, toileting and dressing should be recorded (R1, 97%). 2.6. At onset of the period of NWB, a personalised plan based on the above assessments of where the above care should be delivered should be recorded (R1, 97%). 2.7. During the period of NWB, access to equipment and professional input should be sufficient to deliver care as defined by 1.1–1.2 and 2.1–2.6 and to plan 2.8–2.11 (R1, 100%). 2.8. By the end of the period of NWB, a personalised programme of activity and exercise and where it should be conducted should be recorded (R1, 99%). 2.9. By the end of the period of NWB, plans for the monitoring and management of any aspect of fracture / injury care (wound, fixation device or limb casting) should be recorded (R1, 100%). 2.10. By the end of the period of NWB, plans for addressing on-going personal and instrumental ADL limitations should be recorded (R1. 100%). 2.11. By the end of the period of NWB, plans for addressing on-going pain should be recorded (R1. 99%). 2.12. By the end of the period of NWB, plans for management of osteoporosis should be recorded (R1,96%). 2.13. All care plans listed above should be developed with the patient, with a family member or caregiver if requested by the patient or in those patients lacking sufficient mental capacity to do so (R1,99%)

**Table 2:** Distribution of panellists across disciplines

Specialty	Round 1 (n)	Round 2 (n)
Geriatric medicine consultants	34	23
Orthopaedic surgeon consultants	5	5
Registered nurses	14	11
Clinical specialist physiotherapists	39	30
Clinical specialist occupational therapists	13	13
Clinical dieticians	5	5
Pharmacists	5	3
Total	115	90

Statements that did not achieve consensus in survey round 1 were:

- Vitamin D supplementation should be offered to all patients without checking Vitamin D levels (33% agreement)
- Nutritional supplements (e.g. Fortisips) should be routinely offered to all patients (40% agreement).
- Protein supplements should be routinely offered to all patients (30% agreement).
- Calorie supplements should be routinely offered to all patients (25% agreement)
- Multivitamin supplements should be routinely offered to all patients (32% agreement)

Panellists did not agree with survey round 1 statement that routine nutritional (either calorie, protein or vitamin) supplementation should be offered to this patient group, largely on the basis that there was insufficient evidence for such an approach, and that doing so was contrary to usual practice. However, panellists commented that nutritional issues were of importance. Based on panellists’ free text responses to survey round 1, the statements we designed for survey round 2 explored whether nutritional supplementation should be individualised or whether there are clear sub-groups in whom supplementation should be routine, such as sarcopenia, frailty or low BMI. As shown in Table 1, there was consensus for individualised nutritional assessment prior to supplementation. The three statements that did not achieve consensus after survey round 2 were:

- Protein supplementation should be offered to patients with frailty, even if a clinical assessment indicates that their needs can be met by dietary means (43% agreement)
- Protein supplementation should be offered to patients with sarcopenia, even if a clinical assessment indicates that their needs can be met by dietary means (48% agreement)
- Protein supplementation should be offered to patients with low BMI, even if a clinical assessment indicates that their needs can be met by dietary means (46% agreement)

## Discussion

We produced 24 statements that achieved consensus drawing from 90 panellists, representing seven clinical disciplines, who had experience in the management of patients with weight bearing restrictions after lower limb fractures.

Our findings represent the first consensus exercise conducted in this field. A strength of this study includes the large sample size that reduces the likelihood of agreement appearing by chance. Our panellists were largely geriatricians and physiotherapists and, in common with most consensus studies where recruitment and retention biases inevitably occur, care must be taken not to assume that statements achieving consensus by our definition imply that there is universal agreement, across all settings and clinical disciplines. Further work to develop a clinical guideline should be inclusive of all relevant parties, including patients and their families. Nevertheless, we believe that these statements and the findings of our literature review provide an adequate foundation for the development of a clinical guideline. We appreciate that a clinical guideline based mainly upon expert opinion rather than higher levels of evidence will require subsequent demonstration and affordability studies, especially if the actions implied by these statements are unfamiliar to clinicians or require additional resource provision to deliver.

Our findings are complementary to existing guidelines on the management of fragility fractures and osteoporosis by advising an orthogeriatric model of care and that these patients have access to a fracture liaison service.

Clinicians managing these patients can use these findings in quality improvement work. Collaborations of professional groups can also use these findings in the development of a clinical guideline, bearing in mind that by their nature consensus statements are not as robust as statements drawn directly from robust clinical trial findings. Researchers can use these findings to optimise care when novel interventions are trialled, and may stimulate further research questions. For example, given the residual uncertainty we found, researchers are also justified in evaluating various nutritional strategies in this patient group, or examining the merits or demerits of checking vitamin D levels before determining whether or how to prescribe vitamin D.

## Conclusions

The consensus study findings that achieved consensus drawing from panellists, representing seven clinical disciplines show promise towards developing a clinical guideline in the management of patients with weight bearing restrictions after lower limb fractures. Further work to develop a clinical guideline should be inclusive of all relevant parties, including patients and their families. Still, clinicians managing these patients can use these findings in quality improvement work. Additionally, researchers can use these findings to optimise care when novel interventions are trialled and may stimulate further research questions.

## Supplementary Information

**Additional file 1.**

## Data Availability

All data generated or analysed during this study are included in this published article and its supplementary information files.

## Abbreviations

NWB: Non weight bearing
PPI: Patient and Public Involvement
NHS: National Health Services
JISC online survey: Joint Information Systems Committee online survey
RI: Round 1
R2: Round 2
ADL: Activities of Daily Living
n: Sample size
BMI: Body Max Index
NIHR: The National Institute for Health Research
