# Supplementary material for: Optimal care for the management of older people non-weight bearing after lower limb fracture: a consensus study
Source: BMC Geriatr. 2021 May 24;21:332. doi: 10.1186/s12877-021-02265-z (PMC8146625; doi:10.1186/s12877-021-02265-z)
Supplement: Supplementary file 1 — Additional file 1. [file 12877_2021_2265_MOESM1_ESM.docx]

**Supplementary data: APPENDIX 1**

Professional organisations contacted

Geriatric medicine consultants were approached through:

- - the British Geriatrics Society (<https://www.bgs.org.uk>)
  - the Falls and Bone Health Special Interest Group (<https://www.bgs.org.uk/falls-and-bone-health>)
  - European Geriatrics Community ([https://www.eugms.org](https://www.eugms.org/))
  - the European Falls and Fractures Special Interest Group ([https://www.eugms.org/research-cooperation/special- interest-groups/falls-and-fractures.html](https://www.eugms.org/research-cooperation/special-interest-groups/falls-and-fractures.html)).

Orthopaedic surgery consultants consultants were approached through:

- - the Royal College of Surgeons (<https://www.rcseng.ac.uk>)
  - Orthopaedic Surgeon Special Interest Group (https://[www.abhi.org.uk/who-we-are/member-groups/special-interest-](http://www.abhi.org.uk/who-we-are/member-groups/special-interest-) sections/orthopaedics/)
  - Fragility Fracture Network (https://www.fragilityfracturenetwork.org) and The Orthopaedic Trauma Society ([https://www.orthopaedictrauma.org.uk](https://www.orthopaedictrauma.org.uk/)).

Registered nurses were approached through:

- - Royal College of Nursing https: (<https://www.rcn.org.uk/>)
  - British Nurses’ Association ([https://www.bna.co.uk](https://www.bna.co.uk/))
  - Society of Trauma Nurses ([https://www.traumanurses.org](https://www.traumanurses.org/))
  - The Society of Trauma Nurses – Europe (https://[www.traumanurses.org/about/stn-europe).](http://www.traumanurses.org/about/stn-europe))

Clinical specialist physiotherapists were approached through:

- - the Chartered Society of Physiotherapy(<https://www.csp.org.uk>
  - special interest groups such as Association of Chartered Physiotherapists in Orthopaedic Medicine and Injection Therapy (<https://acpomit.csp.org.uk>)
  - Chartered Physiotherapists Working with Older People and Association of Trauma (<https://agile.csp.org.uk>)
  - Orthopaedic-Chartered Physiotherapists (https://atocp.csp.org.uk )
  - the European Region of the World Confederation for Physiotherapy([https://www.erwcpt.eu](https://www.erwcpt.eu/)).

Clinical specialist occupational therapists were approached through:

- - Royal College of Occupational Therapy(https://www.rcot.co.uk)
  - special interest groups such as the Occupational Therapy for elderly group (https://[www.rcot.co.uk/about-](http://www.rcot.co.uk/about-) us/specialist-sections/older-people-rcot-ss)
  - Trauma and Musculoskeletal Health work group ([https://www.rcot.co.uk/about-us/specialist-sections/trauma-and- musculoskeletal-rcot-ss/clinical-forums](https://www.rcot.co.uk/about-us/specialist-sections/trauma-and-musculoskeletal-rcot-ss/clinical-forums))
  - Council of Occupational Therapists Association for the European Countries (https://www.coteceurope.eu).

Clinical dieticians were approached through:

- - the British Dietetic Association (<https://www.bda.uk.com>)
  - the Academy of Nutrition and Dietetic <https://www.eatright.org>).
  - the European Federation of the Associations of Dietitians (<http://www.efad.org/en-us/about-efad/>

Pharmacist were approached through:

- - The General Pharmaceutical Council (<https://www.pharmacyregulation.org>)
  - the United Kingdom Clinical Pharmacy Association (<https://www.ukclinicalpharmacy.org>)
  - the European Association of Hospital Pharmacists (<https://www.eahp.eu>)
  - the Pharmaceutical Group of European Union (https://www.pcne.org).

**UK NHS Hospitals Trusts contacted**

- Nottingham University Hospitals NHS Trust
- Nottinghamshire Healthcare NHS Foundation Trust
- Birmingham and Solihull Mental Health NHS Foundation Trust
- Birmingham Community Healthcare NHS Foundation Trust
- Birmingham Women's and Children's NHS Foundation Trust
- Derbyshire Community Health Services NHS Foundation Trust
- Derbyshire Healthcare NHS Foundation Trust
- East Suffolk and North Essex NHS Foundation Trust
- East Sussex Healthcare NHS Trust
- Lancashire & South Cumbria NHS Foundation Trust
- Lancashire Teaching Hospitals NHS Foundation Trust
- Norfolk and Norwich University Hospitals NHS Foundation Trust
- Norfolk and Suffolk NHS Foundation Trust
- Oxford Health NHS Foundation Trust
- Oxford University Hospitals NHS Foundation Trust
- St George's University Hospitals NHS Foundation Trust
- The Queen Elizabeth Hospital, King's Lynn. NHS Foundation Trust

**Supplementary data: Appendix 2. Survey Initial statements**

1. statements about Generic
   1. Optimal care for the management of older people with frailty, non-weight bearing after lower limb fracture should comply with current national guidelines for the care of patients with fragility fractures with respect to osteoporosis detection and management; falls risk reduction, and nutrition.
   2. Irrespective of the place of care, patients require access to a multi-professional team including orthopaedic surgeons; and physicians, nurses and rehabilitation professionals with expertise in geriatric care. They will take an approach based on, or compatible with, comprehensive geriatric assessment – covering 1.3a symptoms 1.3b physical functioning 1.3c continence 1.3d activity – previous and current, personal and instrumental ADL 1.3e management of co-pathology beyond osteoporosis 1.3f skin integrity 1.3g a medication review 1.3h cognition 1.3i affect 1.3j social network 1.3k environment 1.3l personal factors (e.g. religious or cultural needs/ requirements).
   3. Inpatient care for these patients should be via an orthogeriatric service (hip fracture care).
   4. Osteoporosis management and falls risk reduction should be co-ordinated by a fracture liaison service (other fragility fractures).
   5. Vitamin D status should be checked, and replacement offered to those who are deficient.
   6. Vitamin D supplementation should be offered to all patients without checking Vitamin D levels.
   7. The daily requirements for protein, calories, vitamins and other vital nutrients for each individual patient should be estimated, and a dietary plan to meet those requirements produced.
   8. Nutritional supplements (e.g. fortisips) should be routinely offered to all patients in addition to 1.8.
   9. Protein supplements should be routinely offered to all patients in addition to 1.8.
   10. Calorie supplements should be routinely offered to all patients in addition to 1.8.
   11. Multivitamin supplements should be routinely offered to all patients in addition to 1.8.
2. Specifically related to the period of non-weight bearing
   1. At the onset of the period of NWB, a personalised programme of activity and exercise should be devised, agreed and recorded: 2.1a to reduce sedentary behaviour 2.1b to include a daily range of motion exercises for the lower limb joints 2.1c to include daily aerobic fitness exercises 2.1d to include daily strength exercises for all limbs.
   2. At onset of the period of NWB, thromboembolism prevention management should be reviewed, and should comprise 2.2a mobilization 2.2b mechanical (e.g. compression hosiery if tolerated) 2.2c low dose heparinoid unless contraindicated.
   3. At onset of the period of NWB, plans for the monitoring and management of any wound, fixation device or limb casting during the period of non-weight bearing should be recorded.
   4. At the onset of the period of NWB, plans for the duration of the period of non-weight bearing, or the decision-making process to define it, should be recorded.
   5. At the onset of the period of NWB, specific plans for the consequences of the personal ADL limitations imposed by the requirement for non-weight bearing such as upon skincare, continence, toileting, and dressing should be recorded.
   6. At onset of the period of NWB, a personalised plan based on the above assessments of where the above care should be delivered should be recorded.
   7. During the period of NWB, access to equipment and professional input should be sufficient to deliver care as defined by 1.1-1.2 and 2.1-2.6 and to plan 2.8-2.11.
   8. By the end of the period of NWB, a personalised programme of activity and exercise and where it should be conducted should be recorded.
   9. By the end of the period of NWB, plans for the monitoring and management of any aspect of fracture / injury care (wound, fixation device or limb casting) should be recorded.
   10. By the end of the period of NWB, plans for addressing on-going personal and instrumental ADL limitations should be recorded.
   11. By the end of the period of NWB, plans for addressing on-going pain should be recorded.
   12. By the end of the period of NWB, plans for management of osteoporosis should be recorded.
   13. All care plans listed above should be developed with the patient, with a family member or caregiver if requested by the patient or in those patients lacking sufficient mental capacity to do so.

**Supplementary data: Appendix 3.** Names of panellists who completed round 2 (n=90).

Aideen Ryan, Alex Trompeter, Amanda Baker, Bruce Love, Carl Bergman, Caroline Jagger, Catherine Barr, Ceri McEwan, Charlotte Abrahamsen, Cherry Crawshay, Chris Potter, Christine Davison, Clare Bostock, Dale Avers, Daniel Bailey, David Keene, David Scott, Devorah Spiegel, Jane Youde, Eleanor Hammond, Elisabete Roldão, Emmanuel Abeka, Esa Jämsen, Frances Sommerville, Frede Frihagen, Gaurav Singh, Gemma Knight, Giuseppe Bellelli, Gülistan Bahat, Hanna Pajulammi, Hannah McGinty, Hollie Pascoe, Iain Wilkinson, Ian Chan, Issy Douglas, Jacqui Close, James Rushton, Jane Fletcher, Jane Hawes, Jane Young, Joe Middleton, John Dow, Jonathan Treml, Judith Randall, Jyoti Shenoy, Katherine Dean, Katie Lockhart, Kelly Tang, Laura Holdway, Laura Salter, Lesley Smith, Louise Hobley, Maggie McDowell, Mairead Groarke, Marco Bravi, Marco Invernizzi, Maria Lucey, Maria Charumbira, Marion Croft, Mark Baxter, Martin Vernon, Maureen Godfrey, Michelle Day, Patricia McIlwaine, Peter Fare, Philip Mathew, Pippa Collins, Pishtiwan Kalmet, Rachel Lyon, Rene Gray, Rene van Mierlo, Ruth Freeman, Sally Sampson, Sam Stirling, Sarah James, Shinta Nishioka, Stacey Finlay, Steffi Bailey, Stephen Allsup, Steven Geldart, Suzanne Swaine, Terence Ong, Tina Coe, Tom Challinor, Victoria Campbell, Vikk Howard, Vishal Kumar, W Kieffer, Wender Figved, Yannis Dionyssiotis.
